# The Retrospective Address in Surgery, from July 1836, to July 1839, Delivered before the Meeting of the Provincial Medical Association, at Liverpool, on the 24th July

**Published:** 1840

**Authors:** 


The Retrospective Address in Surgery, from July 1836, to July 1839, delivered before the Meeting of the Provincial Medical Association, at Liverpool, on the 24th July, and published in the Eighth Volume of its Transactions. By *J. H. James*, Esq. Surgeon to the Devon and Exeter Hospital. 8vo. pp. 92.

Mr. James has published this Address, which gives an account of what has been done in surgery for the last three years. He follows in the wake of Mr. Crosse. The Address does not require any lengthened notice from us, as most of what he states may be found in our own pages. We need only allude to a fact or two, and an opinion.

*Weight and Pulley for Fractures.*—" M. Josse, of Amiens, has proposed a plan of treating these fractures by permanent extension, and in a few words it may be stated to consist in fixing the foot to the bottom of the bed, which is raised, so that the weight of the head and trunk being depending, produce a constant extension along the plane. To this some inconveniences must attach, although they are not much regarded by the proposer. I may perhaps be permitted to say that, nearly two years before M. Josse's work was put into my hands, I had adopted the same principle, but carried it into effect in a different, possibly it may be thought a preferable mode, viz., by fixing the superior part of the body to the head of the bed, which is raised, and making extension on the limb by means of a weight fixed to the leg, properly guarded, and acting over a pulley, assisted also by a very simple apparatus, which it would occupy too much time to describe here." 20.

We would observe that it has long been customary to employ a weight and pulley to extend the limb in cases of threatened consecutive luxation of the femur from disease of the articulation. We cannot say that we ever saw much good in *that* case.

*Necrosis.*—" The difficulty of penetrating the hardened case of new bone when long formed, is too well known to require any comment; and it not unfrequently happens that any attempt to reach the sequestrum is either rendered abortive thereby, or occasions such a degree of disturbance to the whole shaft, as to produce more harm than good. Mr. Guthrie, to whom I allude, has availed himself of the peculiar properties of a remedial agent recently introduced (to which I shall again have occasion to refer)—the chloride of zinc, which, attacking the animal tissue of the bone, destroys it, and thus causes the earthy matter to soften and become detached. The sequestrum is by this means exposed with little pain or disturbance of the part, and may be dealt with according to circumstances. To the success of this plan I can myself most willingly testify." 43.

*New Terms in Medicine.*—" Another topic to which I feel it my duty to advert, is the remarkable fondness for the introduction of new terms in every department of medical science. Without for a moment questioning the propriety of abandoning those which manifestly involve an error, there were many free from any objection of this kind, because, purely arbitrary, and it would have been safer and perhaps better to retain them than to adopt others founded on scientific discoveries, in some cases questionable and liable, like their predecessors, to be reversed. Those who have witnessed with sufficient attention the repeated alterations which have occurred in our own times, will smile at the confidence now expressed in the immutable character of the technology of the day. A change of terms must be a positive good or a great evil. Amid the multiplicity of matters which engross our attention, it is very possible that confusion may arise, for we have the difficult task of unlearning what it has cost us some pains to acquire, and of learning that which is liable to be indistinctly impressed on our minds, just as one sign painted over another is often imperfectly portrayed. If new terms however are to be introduced, it would be well if they were checked by some competent authority, and not be thrown out the unlimited issue of individual speculation. For this evil I see no remedy at present except in the general jealousy of the profession, but that ought to be exercised to keep the prevailing spirit within moderate bounds." 81.

The Address is characterised throughout by good sense.

